# Intersecting paths: exploring the link between neurodevelopmental disorders and chronic pain

**DOI:** 10.1192/j.eurpsy.2025.246

**Published:** 2025-08-26

**Authors:** O. Kilic

**Affiliations:** Department of Neurosciences, Bezmialem Vakif University, Institute of Health Sciences, Istanbul, Türkiye

## Abstract

**Abstract:**

Individuals with neurodevelopmental disorders, such as ADHD and autism spectrum disorder, often experience higher rates of chronic pain compared to the general population. Potential shared mechanisms could be central sensitization, muscular dysregulation and altered pain experiences. Individuals with ADHD and chronic pain frequently have a lower health-related quality of life, including higher pain interference and depressed symptoms. Psychological inflexibility, insomnia and hypermobility are identified as mediators in the relationship between neurodevelopmental symptoms and chronic pain. Understanding these connections can aid in developing targeted interventions to improve the quality of life for individuals with neurodevelopmental disorders experiencing chronic pain.

**Disclosure of Interest:**

None Declared

